# SLINGER: large-scale learning for predicting gene expression

**DOI:** 10.1038/srep39360

**Published:** 2016-12-20

**Authors:** Kévin Vervier, Jacob J. Michaelson

**Affiliations:** 1 University of Iowa, Carver College of Medicine, Department of Psychiatry, Iowa City, 52242, USA

## Abstract

Recent studies have established that single nucleotide polymorphisms are sufficient to build accurate predictive models of gene expression. Gamazon, *et al*., found that gene expression values predicted from cis neighborhood SNPs show statistical association with disease status. In this work, we remove the cis neighborhood constraint during the learning process, and propose a novel predictive approach called SLINGER. We demonstrate that models drawing from a genome-wide set of SNPs are able to predict expression for more genes than the ones built on cis neighborhood only. Results indicate that these new models significantly improve accuracy for a large number of genes. Thanks to a penalized linear model, we also show that the number of features used in our models remains comparable to the cis-only models. Finally, SLINGER application on seven Wellcome Trust Case-Control Consortium genome-wide association studies demonstrate that compared to a cis-only approach, our models lead to associations with greater fidelity to actual gene expression values.

In the last ten years, genome-wide association studies (GWAS) have helped to uncover links between genetic variants and complex diseases. However, these associations are generally difficult to demonstrate statistically, given the lack of power of single-variant tests. Current approaches using polygenic risk scores^1^,^2^ have been developed to robustly aggregate variants, such as single nucleotide polymorphisms (SNPs). Still, these combinations of variants often lack biological interpretability. Recently, Gamazon, *et al*. proposed a new aggregation approach, PrediXcan^3^, which uses gene expression as an intermediate phenotype between genotype and a given trait. This method relies on building a predictive model trained to infer a given gene expression based on a SNP dosage matrix, containing more than 700,000 variants that were used as input features. To reduce the computational burden, a feature selection approach, Elastic Net^4^, was used on a restricted search space of the cis-SNPs belonging to a 1 Mb neighborhood around a given gene. This way, model fitting would be less computationally intensive, but would also leave ~95% of the available data unused. Moreover, although cis-expression quantitative trait loci (eQTL) are more prevalent, it is well-known that trans-eQTL can have large associated effects with disease traits^5^. From a statistical learning perspective, feature pre-filtering is a common way to reduce the dimensionality of a problem. However, it has been observed that this can be detrimental when employing penalized models^6^, such as Elastic Net. Previous results^7^ also suggest that the feature selection that occurs during the training of a penalized model is more robust than a manually derived selection.

In this study, we evaluate gene expression inference using Short and Long-range INfluencers of Gene Expression Regulation (SLINGER). These models were trained on the set of all 719,061 SNPs genotyped in the Depression Genes and Networks (DGN) dataset^8^. We demonstrate that on this data set, SLINGER performs better than state of the art PrediXcan, for a large set of genes. Interestingly, we train 2,267 additional gene expression models for genes that were not estimable at all with PrediXcan, enabling the discovery of trait associations for a much larger set of genes. Even while drastically increasing the number of available features, we observe that on average, our predictive models employ less than 50 predictive SNPs. Furthermore, our results suggest that, for a vast majority of genes, robust predictive signal is contained outside the cis-neighborhood. Finally, applications to Wellcome Trust Case-Control Consortium (WTCCC) GWAS data sets show that statistical associations between disease trait and predicted gene expression values exhibit significantly increased fidelity to measured gene expression (as measured by *r*^2^) with the SLINGER approach, and these improvements lead to novel gene-phenotype associations.

## Results

### Models training on DGN data

**Figure 1:**
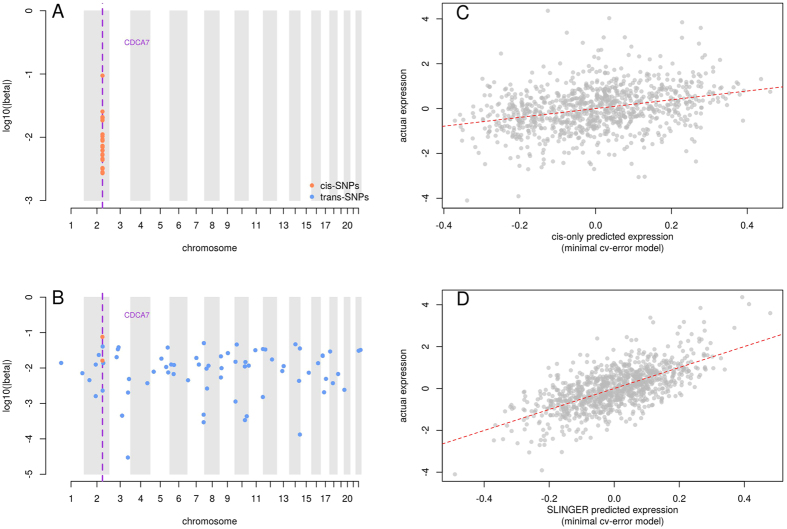
Comparison of properties between a model trained on constrained set of cis-SNPs and a SLINGER model trained using all available SNPs. Performance metrics are reported for CDCA7, found to be significantly associated with Type 1 diabetes. (**A**) Genome-wide positions of SNPs used in the cis-only model. (**B**) Genome-wide positions of SNPs used in the SLINGER model. In both left figures, orange dots represent SNPs found in the cis neighborhood of the gene and the blue dots are trans-SNPs. The purple dashed line corresponds to the gene location. (**C**) Comparison between cross-validated predictions for the cis-only model (x-axis) and actual gene expression found in the Depression Genes and Networks (DGN) data set (y-axis). (**D**) Comparison between cross-validated predictions for the SLINGER model (x-axis) and actual gene expression found in DGN data set (y-axis). In both right figures, each grey dot corresponds to one individual gene expression level, and the dashed red line represents the linear fit between predicted and actual gene expression.

**Figure 2:**
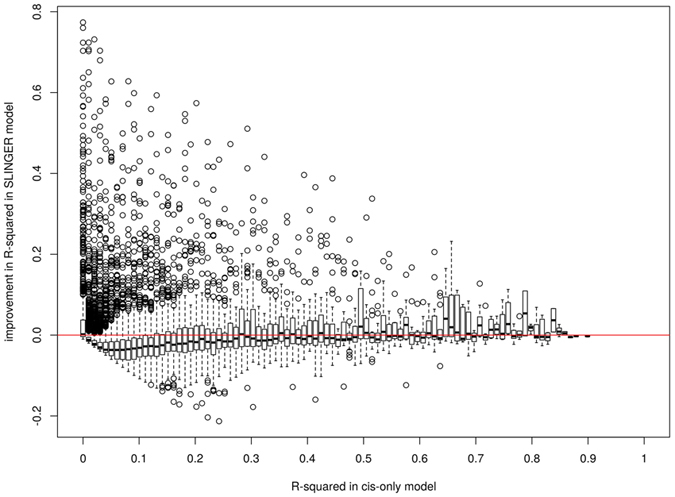
Gene-level gain in accuracy with unrestricted SLINGER models. We report model performance for 13,825 genes present in the Depression Genes and Networks (DGN) dataset. *r*^2^ measures for PrediXcan models are binned in 101 subsets along x-axis. The difference between *r*^2^ values for SLINGER and PrediXcan models are represented on the y-axis. The red line corresponds to a no-gain case and everything above represents a gain when the unrestricted model is used.

We used data from 922 whole-blood samples from the Depression Genes and Networks^8^ (DGN) study to train predictive models (see Methods). Dosages for all SNPs characterized in this study were used as the input set of features, with no preliminary filtering based on, for instance, minor allele frequency (MAF). Furthermore, where PrediXcan utilized imputed genotypes, we trained our models only on observed SNPs. Considering the whole set of available SNPs in the DGN data allows us to train 2,267 additional gene expression models for genes that are not estimable using only SNPs in the cis-neighborhood (see list in Supplementary Table 1). Also, we report in Supplementary Table 2, 1,866 genes for which SLINGER models perform at least 10% better than PrediXcan models in terms of *r*^2^. We represent, in Fig. 1, an example comparison between the two approaches, both in terms of selected features and cross-validated accuracy. Figure 2 shows the overall gene-level gain in accuracy with unrestricted SLINGER models, compared to cis-only models provided by PrediXcan, for 13,825 genes. During the training of SLINGER models, we consider a set of descriptive features approximately 20 times larger than the ones using only the cis-SNPs. cis-SNP predictive models, like PrediXcan, used genotype information found in a ±1 Mb neighborhood of the predicted gene boundaries. On average, this translates to around 35,000 available features for each predicted gene. The average number of features with non-zero weight used in a cis-only model is 14.28 ± 0.285, compared to 41.87 ± 1.04 for SLINGER models. Interestingly, although the number of features available is far greater, the number of selected features in SLINGER scales in a sub-linear fashion with respect to the number of features in the cis-only model (*N*_*all*_ = 0.27 × *N*_*cis*_ + 39.6). This suggests that the feature selection induced by Elastic Net is sufficient to filter out a vast majority of the non-associated predictors. In terms of computational burden, SLINGER models only show a marginal increase of 10% of prediction time (~13 seconds for 8,000 genes prediction).

**Figure 3:**
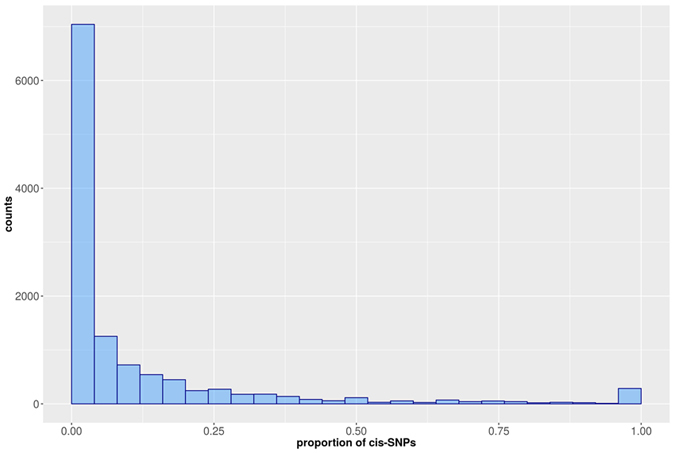
Proportion of cis-SNPs used to train SLINGER models. For each of the 11,958 genes predicted by unrestricted models, the proportion of selected features found in the cis-neighborhood is reported. A large proportion (41.2%) of SLINGER models do not use any cis-SNP information, meaning that they rely on signals contained in locations far from the gene position (e.g., trans-effects).

Figure 3 represents the complete distribution of the proportion of cis-SNPs used in models and illustrates that 41.2% of unrestricted models do not use any cis-SNP information, meaning that they rely on signals contained in locations far from the gene position (e.g., trans-effects).

### Application to WTCCC GWAS data sets

**Figure 4:**
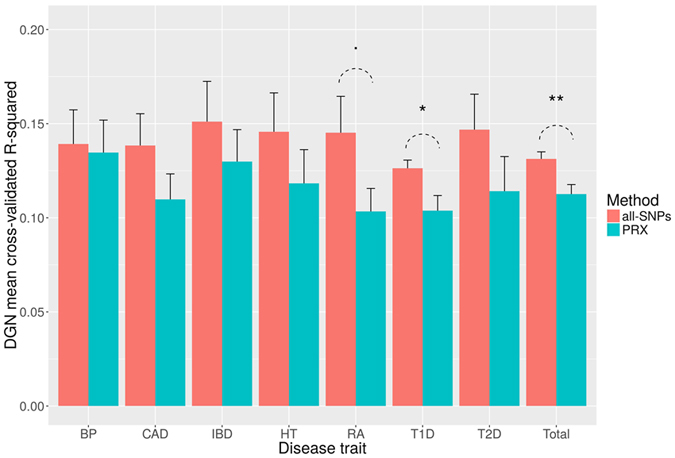
Gene-Phenotype associations for seven WTCCC GWAS data sets. For each trait, we reported the average cross-validated *r*^2^ on associated genes, for both SLINGER (red) and PrediXcan (blue), obtained on the Depression Genes and Networks (DGN) data set. Significance between the two approaches has been tested using Student t-test (‘·’p-value < 0.1, ‘*’p-value < 0.05, ‘**’p-value < 0.01). BP, bipolar disorder; CAD, coronary artery disease; IBD, inflammatory bowel disease; HT, hypertension; RA, rheumatoid arthritis; T1D, type 1 diabetes; T2D, type2 diabetes.

We applied PrediXcan and SLINGER predictive models to seven diseases characterized in WTCCC studies^9^, namely bipolar disorder, coronary artery disease, inflammatory bowel disease, hypertension, rheumatoid arthritis, type 1 and type 2 diabetes. For each disease, we first estimated expression for 11,958 genes using both approaches. We then used logistic regression for testing the statistical association with disease status, and used a Bonferroni-corrected threshold to keep significant hits only. In Fig. 4, we show the accuracy of the expression prediction for associated genes, in terms of average *r*^2^. For each WTCCC trait, our approach yields gene-disease associations with higher DGN cross-validated *r*^2^, and the overall performance is significantly better for SLINGER (p-value = 0.003).

## Discussion

In this work, we present SLINGER, a valuable extension to PrediXcan^3^ for building predictive models linking genotypes to disease traits, via the intermediate phenotype of gene expression. We rely on an unrestricted set of SNPs, shared by all the trained models and demonstrate that this approach 1) increases the number of estimable genes by 2,267 and 2) improves prediction performance for another set of about 2,000 genes. On WTCCC GWAS data sets, we observed that our unrestricted approach led to gene associations that were characterized by significantly elevated *r*^2^ (Fig. 4). This suggests that these associations are more reflective of actual variation in gene expression, and are less likely to be spurious. This is important because the *r*^2^ of a putative association is a key indicator when planning follow-up experiments, as it reflects the degree to which the model predictions track with actual gene expression, and consequently how likely it is that experimental manipulation of the gene’s expression will result in effects relevant to the disease of interest. We also provide a sample of novel gene-disease associations in Table 1. Particularly noteworthy is that SLINGER was able to yield associations for coronary artery disease, a condition where no association was reported in PrediXcan^3^. Specifically, we find significant association for desmoplakin (DSP), which has been robustly implicated in other cardiovascular diseases, such as heart failure^10^, epicardial ventricular tachycardia^11^ and cardiomyopathy^12^. A complete list of gene-disease associations is included in Supplementary Table 3. Given that some genes still have better prediction accuracy with PrediXcan, we recommend a combined approach, where PrediXcan is used for the genes where it achieves optimal performance, and SLINGER models for genes we reported in Supplementary Tables 1 and 2. The present predictive models have been trained on genotype data from microarray technology, resulting in a large but still incomplete set of SNPs. Future work would involve using whole genome sequencing as a way to access predictive features not constrained to a subset of the genome. We also plan to extend our predictive model from whole blood to other tissues (e.g., brain), using tissue-specific databases provided by, for instance, the GTEx Project. Although the current PrediXcan models (trained on whole blood) have already been validated on other tissues beyond whole blood, the potential gain of using a model trained on tissue-specific data compared to a general model remains unclear. Specifically, applying tissue-specific models corresponding to disease-related tissue, for example brain tissue for bipolar disorder, would be expected to improve genotype-phenotype association results appreciably.

## Methods

### DGN RNA sequencing dataset

We obtained whole-blood RNA-seq and genome-wide genotype data (719,061 SNPs) for 922 individuals from the the Depression Genes and Networks (DGN) cohort^8^, all of European ancestry. For our analyses, we used the HCP (hidden covariates with prior) normalized gene-level expression data for 15,231 genes used for the trans-eQTL analysis in Battle, *et al*.^8^ (downloaded from the National Institute of Mental Health (NIMH) repository). We converted the raw genotype format to dosage format, by using PLINK^2^.

### PrediXcan models

We downloaded “DGN Whole Blood Elastic Net” models, based on the imputed DGN data^8^, from the PrediXcan project website (http://github.com/hakyimlab/PrediXcan) and used the available evaluation scripts. For the 3,651 genes that were not estimable by PrediXcan models, we considered their score equal to 0. Note also that we used the same rule for genes not estimable by our models.

### SLINGER models using Elastic Net on genome-wide genotype

To train each SLINGER gene-level model, we rely on regularized least-squares regression:


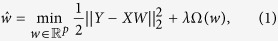


where 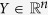 denotes the expression level for a given gene through *n* individuals, 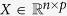 is the corresponding genotype matrix which contains *p* genotyped SNPs, and *w* represents the weights of the predictive model. In our study, SLINGER models rely on *p* = 719,061 genotyped SNPs as input features, whereas in the PrediXcan approach, *p* varies for each gene, based on its cis-neighboorhood content. The function Ω, also called regularizer, usually applies constraints to the model *w*, such as restricting the size of the weights or limiting the number of features with non-zero weights. The parameter *λ* > 0 controls the trade-off between how good the fit is and how constrained the model *w* will be. In this study, we used Elastic Net^4^, where 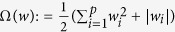. It combines the 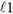 and 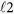 norms to ensure the joint selection of the correlated features, which is a desirable property given the potential trans-effects observed in gene expression data. All gene-level models are publicly available (http://github.com/kevinVervier/SLINGER) and are made compatible with PrediXcan tool.

### Performance indicator

We follow a method evaluation similar to Gamazon, *et al*.^3^. For every gene expression regression model, we compute the coefficient of determination, *r*^2^, provided by the formula


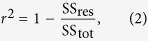


where SS_res_ is the residual sum of squares, and SS_tot_ is the total sum of squares. An *r*^2^ of 1 refers to a perfect fit to the data, where an *r*^2^ equal to 0 means that the model does not fit the data at all.

## Additional Information

**How to cite this article**: Vervier, K. and Michaelson, J. J. SLINGER: Large-scale learning for predicting gene expression. *Sci. Rep.* **6**, 39360; doi: 10.1038/srep39360 (2016).

**Publisher's note:** Springer Nature remains neutral with regard to jurisdictional claims in published maps and institutional affiliations.

## Supplementary Material

Supplementary Tables

## Figures and Tables

**Table 1:** Gene-Phenotype associations for seven WTCCC GWAS data sets, for genes with a higher *r* ^2^ with SLINGER than with PrediXcan.

Disease	Gene	Description	Cross-validated *r*^2^	Citations
CAD	DSP	Desmoplakin	0.68	24
RA	MICA	MHC class I polypeptide	0.71	6
T1D	MICA	MHC class I polypeptide	0.71	13
T1D	COL11A2	Collagen, type XI, *α*2	0.29	1
T1D	SLC22A1	Solute carier family 22 1	0.21	1

We only report significant findings with at least one PubMed co-citation involving the gene and trait, and a cross-validated *r*^2^ higher than 0.1.
